# A Case of Tick-Borne Paralysis in a Traveling Patient

**DOI:** 10.1155/2019/3934696

**Published:** 2019-06-27

**Authors:** Kevin Ha, Kathryn Lewis, Vandan Patel, Jennifer Grinceri

**Affiliations:** University of California, Riverside, School of Medicine, USA

## Abstract

**Background:**

Tick paralysis is a neurotoxic tick-borne illness that causes ascending paralysis and may lead to respiratory failure. Patients often undergo extensive testing and prolonged hospitalization before the proper diagnosis is reached.

**Case Presentation:**

An 88-year-old man with dementia and dyslipidemia presented with new onset gait instability and was admitted for suspected cerebellar stroke. Exam was significant for the inability to perform tandem gait. Investigations included comprehensive metabolic panel, complete blood count, and noncontrast CT scan; none of them found any evidence of acute pathology. Two days into admission, a tick with surrounding erythema was found on the patient's left lateral chest during bathing. Dramatic improvement in truncal ataxia was noted following tick extraction.

**Discussion:**

Clinical suspicion of tick paralysis is often low due to the rarity of the condition. Although it is imperative to rule out acute cerebral or cerebellar pathology, a thorough skin examination should be performed on admission in any patient with new onset ataxia and ascending paralysis. This can lead to early diagnosis, conservation of resources, and the avoidance of subjecting patients to invasive testing.

## 1. Introduction

Tick paralysis is a rare neurotoxic response to the envenoming by an adult female tick endemic to North America and the eastern coast of Australia. The most common presentation is an ascending paralysis, preceded by a nonspecific prodromal phase marked by fatigue, fever, and generalized weakness. Full recovery of paralysis is often reported within 24-48 hours of tick removal [1]. Although rare, untreated tick paralysis carries a 10% mortality rate, secondary to respiratory failure. Risks of paralysis developing after tick bite are correlated with milligram of toxin-per-kilogram of patient as well as the time between attachment and tick removal. Therefore, most reports of tick paralysis are of young girls whose long hair provides camouflage for feeding ticks. We present a rare case of tick paralysis in an active elderly male visiting the desert.

## 2. Case Report

An 88-year-old male with dementia, dyslipidemia, and status after cochlear implantation presented to a community hospital after new onset gait instability. The patient was on a road trip from Wisconsin when he developed an inability to walk at dinner after arriving in the Coachella Valley, approximately 100 miles east of Los Angeles. The patient was unable to ambulate and experienced worsening instability in his trunk. History was negative for atrial fibrillation, transient ischemic attacks, and strokes. The patient's only medications were an 81 mg daily aspirin and simvastatin. Given his progressive truncal ataxia, the patient was admitted for suspected cerebellar stroke.

On exam, the patient was alert and oriented to person, place, and time without sensory or cranial nerve deficits. Strength was 5/5 in all limbs. Exam was remarkable for ataxia of the trunk with unstable gait. Patient was unable to perform tandem gait. Laboratory findings for complete metabolic panel and complete blood count were unremarkable. A noncontrast CT was done, revealing stable age-related atrophy without evidence of acute ischemia or hemorrhage. Complete evaluation for cerebellar stroke was limited, as MRI could not be done due to cochlear implantation. The patient was then admitted for further management and workup of ataxia given concern for stroke.

**Figure 1:**
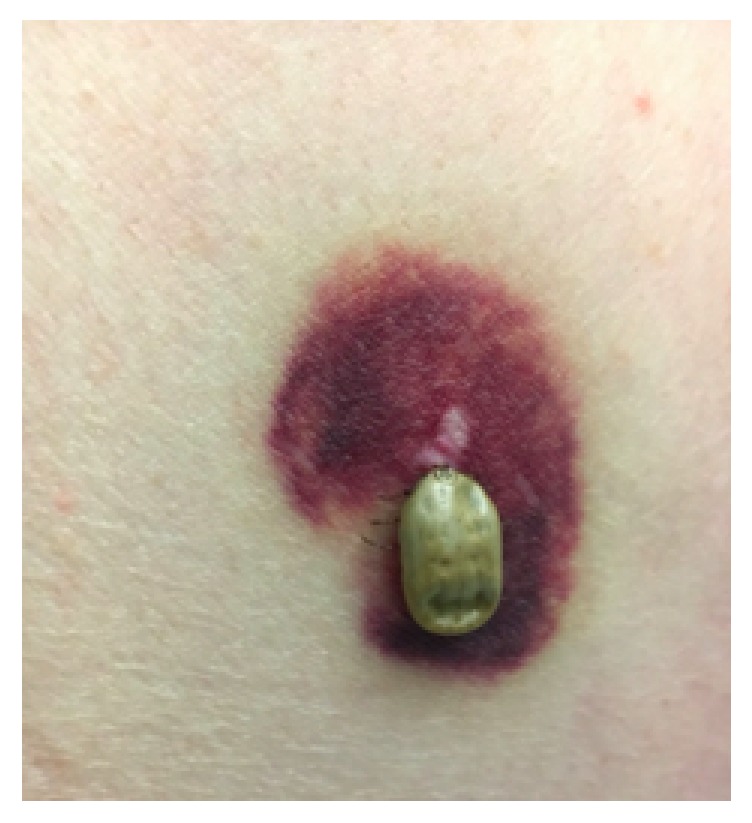
Engorged female tick with surrounding ecchymosis on the left axilla.

**Figure 2:**
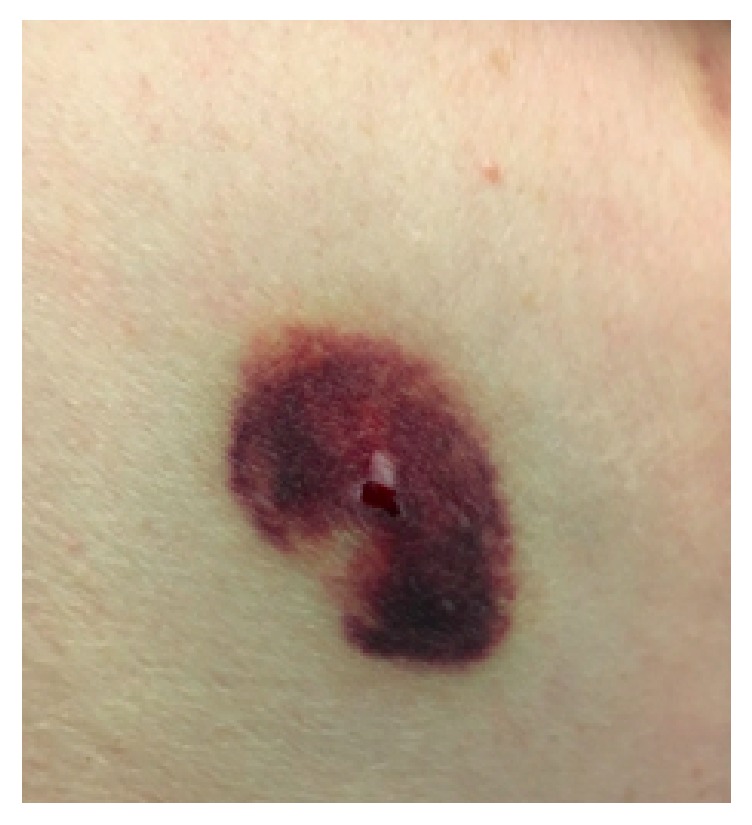
Left axilla ecchymosis status after tick extraction.

Two days into admission, a tick with surrounding ecchymosis was found on the patient's left lateral chest wall during bathing (Figures 1 and 2). Upon further questioning, he reported hotel stays and visited national parks throughout his travels. He denied animal exposures, camping, and use of insect repellant. Following tick removal, truncal ataxia dramatically improved. The patient worked with physical therapy for an additional two days and was then safely discharged without ataxia.

## 3. Discussion

There are over 60 identified species of ticks whose toxins can cause paralysis. The ability to cause paralysis is dependent on the type of tick–soft versus hard, host species, life cycle stage, and sex. For example, the tick identified in this case,* Dermacentor variabilis*, is a hard tick in which the female can only cause paralysis [2]. In the United States, the most reported species is the* Dermacentor *tick, often found in wooded regions of the Rocky Mountains and Pacific Northwest. Ticks often remain dormant under leaves during winter months and become active in the spring, with incidents of tick bites being highest from March to July. Bites are often painless, and ticks may hook onto their hosts for up to ten days [1, 3, 4]. In the process of feeding, neurotoxins from the salivary glands of ticks may be released into the host. It is hypothesized that the toxins produced by ticks, or holocyclotoxin, act similarly to botulinum toxins by blocking the presynaptic release of acetylcholine into the synaptic cleft causing delays to nerve conduction [1, 3–5]; however, there are multiple proposed methods of paralysis unique to different species which may be used in combination or alone [2].

Tick paralysis may be broken up into two distinct stages: prodromal and proper paralysis. The prodromal period often appears within 36 hours and presents with nonspecific flu-like symptoms, headache, and tingling. If the tick remains attached, the proper paralysis phase begins (often by day four) with ataxia followed by weakness to the lower extremities. Eventually, the paralysis ascends, causing lower-motor-neuron signs of flaccid paralysis to all extremities and quadriplegia [1, 3, 4]. Cranial nerve involvement has been reported, with patients presenting with ophthalmoplegia, facial weakness, and bulbar palsy [6]. Although rare, untreated tick paralysis carries a 10% mortality rate, secondary to respiratory failure [1, 3].

Diagnosis of tick paralysis is made via complete physical examination of the skin. Once identified, ticks are removed by grasping the tick as close to the skin surface while avoiding squeezing additional toxins. Full recovery of paralysis has been reported within 24-48 hours of tick removal with repeat nerve conduction study showing amplitude values and conduction time returning to normal [1]. Given that Lyme Disease is caused by the* Borrelia burgdorferi *bacteria transmitted by the* Ixodes scapularis* tick, considerations of travel history to the Northeast US, tick morphology, and symptoms such as target rashes, arthralgia, and heart block should be considered as Lyme Disease requires both tick removal and introduction of oral or IV antibiotics. In this case, we identified the tick causing paralysis to be* D. variabilis *based on geographic predominance [7]. Our group did not attempt to identify the tick species while in the hospital by morphology or direct assay. However, we argue that there is benefit to identifying encountered ticks in future cases for tracking purposes as well as for research that is being done to provide antiparalysis vaccines [2].

More often, tick paralysis is often misdiagnosed as Guillain-Barre Syndrome (GBS), as both diseases present with an ascending paralysis [3]. As a result, clinicians may spend time ordering spirometry, CSF analysis, nerve conduction studies, and intravenous gamma globulin G as a treatment for the ascending paralysis. Interestingly, both tick paralysis and GBS demonstrate similar nerve conduction studies of decreased amplitudes of compound muscle action potentials and diminished nerve conduction velocities [3, 8]. Tick paralysis lacks the fevers or autonomic dysfunction that should point a clinician away from a GBS diagnosis. Other diseases incorrectly worked up include botulism, encephalomyelitis, and stroke [3, 9].

Unfortunately, the literature suggests that detection of ticks in patients presenting with ascending paralysis is often not made during the initial assessment but instead later in the hospital course by the patient's family members, nursing staff, or other providers. This finding is often after an extensive workup for a wide array of diseases was already performed, prolonging the time of attachment to detection and removal of the tick. Given that tick paralysis is dependent on both milligram of toxin-per-kilogram of host as well as time of attachment, delay in care is both an inefficient use of resources and potentially dangerous to patient care [1, 3, 4].

## 4. Conclusion

Tick paralysis is poorly surveilled in the United States with one meta-analysis reporting only fifty well documented cases of tick paralysis between 1946 and 2006 [3]. Patients presenting with symptoms of ascending paralysis in endemic areas or recent travel to endemic areas should prompt clinicians to do a thorough skin examination for ticks before working up other etiologies. A stroke rule-out was both time sensitive and necessary in our patient given his symptoms of acute onset weakness; however, tick paralysis was low on our list of differentials demonstrated by the delay in obtaining the patient's recent travel history and more thorough skin examination. This case further illustrates the importance of proper history taking, physical examination, and consideration of tick paralysis as a differential in patients presenting with ascending paralysis.
